# Assistant Physicians to Provincial Infirmaries

**Published:** 1895-02-09

**Authors:** 


### ASSISTANT PHYSICIANS AND SURGEONS TO PROVINCIAL INFIRMARIES.

Much discussion has taken place at Derby as to a proposed alteration of the rules with regard to the medical staff. The proposal was to create one honorary assistant physicianship and one honorary assistant surgeoncy, and to confine the duties to the charge of the out-patients. In the event of a vacancy on the full staff it was proposed that it should be filled by the election of the assistant physician or surgeon, as the case might be. As the rules are silent on the point, and the qualifications in the case of the assistant surgeon were not raised to a fellowship or degree in surgery, we presume it was intended to appoint someone in general practice to these offices. The whole question of the status of the honorary medical officers of provincial infirmaries was thus involved. Formerly it was found impossible for the honorary medical officers of these institutions to get a living unless they were permitted to engage in general practice. The objections and drawbacks of such a plan have always been patent to the initiated, but unless, or until the population of the county and large provincial towns become alive to the importance of having at least one physician who confines himself to pure medicine, and one surgeon who devotes himself to pure surgery resident amongst them, the hospital governors have no choice but to permit general practice on the part of the members of their staff. Derby now has one of the finest hospitals in the provinces. The institution affords an opportunity of the highest kind for clinical and pathological work. The town of Derby contains nearly 100,000 inhabitants. Hence it is of the first importance in our view to the inhabitants of the town and county of Derby that they should encourage and support at least two physicians and two surgeons, so that all classes may have the benefit of the highest medical service as occasion may arise. We understand, however, that the better class of inhabitants prefer to go to Birmingham, Sheffield, or London when they want a consultant, and so long as this spirit prevails, so long will it be impossible for the governors of the infirmary to alter their rules, and to place the medical work of the infirmary upon the highest clinical and pathological basis. In the existing circumstances we feel constrained to say that the governors of the Derbyshire Infirmary exercised the wisest discretion in declining to create the offices of assistant physician and assistant surgeon. They were wise, in our view, in adopting the alternative course of adding to the staff of the infirmary an extra honorary physician and honorary surgeon. We presume that each of these officers will have the same privileges as to beds and practice possessed by the other physicians and surgeons as those of the infirmary. In making these remarks, we wish it to be understood that we make no reflection whatever upon the present members of the medical staff at the Derbyshire Infirmary, who have so long and so ably borne the burden of attending to the patients who find their way to this great institution. On the contrary, it is but an act of justice that we should urge upon the people of Derby and the district the importance of encouraging the senior members of the staff to gradually relinquish general practice and devote themselves entirely to pure medicine or pure surgery. We congratulate the weekly board upon the temper and spirit which they have displayed throughout the recent discussions, and especially upon their decision not to call in any experts to advise upon the rules, but to work out their own salvation in accordance with what they believe to be possible and best, having regard to local needs and local circumstances.

